# Methodologies in Spectral Tuning of DSSC Chromophores through Rational Design and Chemical-Structure Engineering

**DOI:** 10.3390/ma12244024

**Published:** 2019-12-04

**Authors:** Qudsia Arooj, Gregory J. Wilson, Feng Wang

**Affiliations:** 1 Centre for Translational Atomaterials, Faculty of Science, Engineering and Technology, Swinburne University of Technology, Hawthorn, Melbourne 3122, Australia; qarooj@swin.edu.au; 2 CSIRO Energy, Newcastle Energy Centre, Mayfield West, NSW 2304, Australia; greg.wilson@csiro.au

**Keywords:** organic dye-sensitized solar cells (DSSCs), computer-aided rational design (CARD), HOMO-LUMO energy gap, DFT calculations, UV-Vis spectra

## Abstract

The investigation of new photosensitizers for Grätzel-type organic dye-sensitized solar cells (DSSCs) remains a topic of interest for researchers of alternative solar cell materials. Over the past 20 years, considerable and increasing research efforts have been devoted to the design and synthesis of new materials, based on “donor, π-conjugated bridge, acceptor” (D–π–A) organic dye photosensitizers. In this paper, the computational chemistry methods are outlined and the design of organic sensitizers (compounds, dyes) is discussed. With reference to recent literature reports, rational molecular design is demonstrated as an effective process to study structure–property relationships. Examples from established organic dye sensitizer structures, such as TA-St-CA, Carbz-PAHTDDT (S9), and metalloporphyrin (PZn-EDOT), are used as reference structures for an examination of this concept applied to generate systematically modified structural derivatives and hence new photosensitizers (i.e., dyes). Using computer-aided rational design (CARD), the in silico design of new chromophores targeted an improvement in spectral properties via the tuning of electronic structures by substitution of molecular fragments, as evaluated by the calculation of absorption profiles. This mini review provides important rational design strategies for engineering new organic light-absorbing compounds towards improved spectral absorption and related optoelectronic properties of chromophores for photovoltaic applications, including the dye-sensitized solar cell (DSSC).

## 1. Introduction

Our Sun is an abundant source of free and clean energy and in recent decades has driven the world to develop and improve photovoltaic devices—solar cells—that enable the capture of sunlight and conversion directly to electricity. Although not a recent observation [1], it is well accepted that the Earth receives less than one-billionth [2,3] of the Sun’s energy emissions, yet even that tiny fraction provides the Earth with more energy in one hour than all the energy consumed by humans in an entire year [1]. This has directly influenced the growth of an international solar power industry to manufacture and install solar modules worldwide at record-breaking rates over the past decade. The future of low-cost solar cells, including emerging photovoltaic technologies based on dye-sensitized solar cells (DSSCs), organic compounds, perovskite materials, and quantum dots, are in the spotlight due to their promise as a less expensive alternative that is more adaptable to broader applications than conventional silicon solar cells, which currently claim about 90% of the solar cell market [1,4].

Governed by the principle and “discovery of the law of the photoelectric effect” by Einstein in 1905 [5,6], the phenomenon of the photovoltaic effect was noted over half a century earlier by Becquerel [7] in 1839. Photovoltaic cells, often referred to simply as solar cells, are generally categorized based on the chemistry of the materials the technology utilizes. The earliest solar cells were based on crystalline silicon wafers [8], and the majority of commercially produced residential solar panels currently still rely on this type of material due to their continued development over several decades to achieve a high light-to-electrical power conversion efficiency. However, the relatively high cost of the base silicon material—in terms of the volume of silicon required per wafer and the embodied energy of producing the raw material—has led to the development of more cost-effective “thin-film” solar cells, first emerging in the 1970s. Since then, a focus of research has been to address the significant challenge in developing more cost-effective and higher efficiency solar cells as an alternative to incumbent technologies. To further improve the performance of thin-film photovoltaic (PV) devices, all components require systematic optimization, including new materials, with an emphasis on light-absorption structures to enhance the spectral response and increase overall photon collection properties.

The use of chromophores (i.e., organic light-absorbing compounds or sensitizers) in dye-sensitized solar cells (DSSCs) [9] has received much attention since their inception in the early 1990s. Significant research efforts for such a class of organic dye sensitizers have been carried out for inexpensive alternatives with respect to conventional and expensive photovoltaic devices. Transition metal complexes of *d*^6^ ruthenium dyes based on pyridyl ligands, such as *cis*-bis-diisothiocyanato bis(4,4′-dicarboxylato-2,2′-bipyridine) ruthenium(II) N3 [10,11], di-tetrabutylammonium *cis*-bis(diisothiocyanato) bis(4,4′-dicarboxylato-2,2′-bipyridine) ruthenium(II) N719 [2,3,11], and the so-called black dye, tris(N,N,N-tributyl-1-butanaminium)[[2,2″6′,2″-terpyridine]-4,4′,4″-tricarboxylato]tris(thiocyanato) ruthenium(II) (N749) [12,13,14], are recognized as some of the dye sensitizers for DSSCs with the highest reported performance in terms of their stability and light-to-electrical power conversion efficiency (PCE), which exceeds 11% under AM1.5 test conditions [3,11]. However, these compounds contain the transition metal element, ruthenium, which is a rare element (with an abundance of 100 parts per trillion in the Earth’s crust) and hence relatively expensive elemental compound, whereas the preparation of dyes using organic structures offer almost limitless abundance, translating to a cost-effective alternative. Moreover, organic dyes afford desirable properties, such as tunable optical properties and high absorption coefficients [15,16,17,18,19]. For organic dyes, the most important issue that needs to be addressed is the improvement and/or extension of the relatively narrow spectral absorption properties in comparison to Ru-based complexes [19,20]. This area of research around organic dye structures has attracted increased interest in recent years and broader applications than just solar cells, with a year-on-year growth since 2010. As a result, over 1000 articles were published in 2010, growing to more than 3000 by 2015, as shown in the Web of Science database [21].

An immediate challenge to improving the efficiency of DSSCs is to overcome the design and testing of new materials (e.g., dye sensitizers), which has been dominated by the often costly and time-consuming synthesis procedures [19]. In the processes of experimental development of new dye sensitizer materials, without necessary prior supporting information of the target new dyes, a difficult hurdle for a rapid turnaround in synthetic laboratories is screening for high-performance dyes with desirable absorption properties [22]. For example, it was reported that two chemically similar synthesized dyes [23] differed significantly from the reported energy conversion efficiencies when being fabricated into DSSCs: One compound has an efficiency of η = 6.79% and the other yields an efficiency of η = 4.92% [22]. However, the two dyes differ structurally only in the use of the backbone of π-spacers: One dye utilizes thiophene (η = 6.79%) and the other a thiazole (η = 4.92%) [23]. Both π-spacers have sulphur incorporated in the heterocyclic ring while the latter (thiazole) additionally contains nitrogen in the cyclic ring structure. It is difficult to correlate the structure–property relationships of dyes simply through “chemical intuition”; the mixing of electronic states gives rise to distinctly different optoelectronic properties. As a result, electronic properties obtained through accurate quantum mechanical calculations are essential to provide quantitative information to aid our understanding of the electronic interactions of dyes at the molecular level.

One of the very important components of DSSCs is that the dye sensitizers need to broadly absorb in the visible and near infrared (NIR) fractions of solar irradiation. The absorption of light excites electrons from the ground state of the dye molecule and the excited electrons are then injected to the conduction band of an intimately bound semiconductor surface. The design of dye sensitizers through a pure synthetic approach is time consuming and can be very expensive, with more complex synthetic steps leading to commercial non-viable options [19]. Alternatively, computer-aided rational design (CARD) of organic dye sensitizers becomes increasingly attractive and has rapidly become a knowledge-based state-of-the-art practice in the development of novel organic compounds, including chromophores and dyes in DSSC application [24,25,26,27,28]. The purpose of CARD is to either improve or enhance the desirable properties of organic dye sensitizers, usually through rational chemical substitutions (structural alternative/analogues) of available high-performing dye structures, using high-performance computing (HPC) to engineer new candidate structures. CARD enables us to develop a knowledge-based model, in order to study desirable properties from benchmark high-performance dyes and screen these structures for effectiveness; new candidate dyes can be identified prior to proceeding with synthesis. Such rational design has been widely adopted in other industries as an effective screening tool, particularly in drug development [29,30,31] and other functional materials by design.

A key objective of CARD in the design of light-absorbing dyes and chromophores is to maximize photon absorption through increased red-shift (bathochromic) absorption of the UV-Vis absorption spectrum and to enhance electron transfer through improved electronic overlap of excited state orbitals, effectively shifting the spectrum with respect to a reference dye structure. In order to do so, the energetically most favorable excitation, e.g., π → π* (or n → π*), occurs from the highest energy-bonding π-orbital (HOMO) to the lowest energy-antibonding π*-orbital (LUMO), which serves as the focus for the primary electronic transition. Strategically, this reduction of the HOMO-LUMO energy gap and shifting of the frontier molecular orbitals through chemical structure modification is a first step towards improved structures, under specific conditions for DSSC applications [32]. However, the utilization of differing functional chemical groups and integration within new candidate chromophores (dyes) with respect to a reference dye structure presents a significant challenge; often, chemical intuition is insufficient and the chemistry on various chromophores can be very different. Guidance surrounding general rules for new dye design remains a topic of research interest and investigation.

The visible region of the spectrum comprises photon energies from 36 to 72 kcal⋅mol^−1^ (i.e., 400–800 nm), and the near ultraviolet (UV) region, out to 143 kcal⋅mol^−1^ (i.e., 200 nm). With respect to relevant chemical structure, for common organic dyes, these are a system of extensively conjugated π-electrons. As a result, one of the most popular structures for dye sensitizers in DSSCs is the generic chromophore structure of “donor, π-conjugated bridge, acceptor” (D–π–A) or ‘push-pull’ dye, for instance, the TA-St-CA dye [24]. This structure utilizes a π-conjugated bridge (e.g., oligo-phenylenevinylene) with an electron donor (e.g., the triphenylamine (TPA)-based fragment) and an electron acceptor (e.g., 2-cyanoacrylic acid moiety) to induce a preferred absorption shift in the overall chromophore. It is found that systematic modification of the donor, π-bridge, or the acceptor moieties all serve the purpose of property and spectral manipulation of the original dyes. For example, modifications of the conjugated π-bridge in a push-pull dye can significantly shift the absorption spectrum of the new dyes. If a dye is modified in such a way to maximally absorb the solar energy, it will be able to enhance the efficiency of DSSCs [33]. The application of Dewar’s rules [34] based on molecular orbital theory provides excellent guidance to rational structure–property relationships between the conjugation bridge of chromophores [24].

In this paper, we summarize recent studies in organic DSSCs using CARD and applied in our laboratory. The structural and electronic properties of new light-absorbing dyes designed from several original high-performing dye structures are presented, in which modifications of high-performing dyes, i.e., TA-St-CA dyes [24], Carbz-PAHTDDT (S9) [25], and Zn-Ph dyes [26], will be highlighted. For example, the use of Carbz-PAHTDDT with a TPA-based donor (D) moiety substituted by two carbazole units and dithienothiophene (DTT), a five-membered heterocyclic ring, as a parent structure with a conjugated π-bridge and the acceptor group. Further, Pzn EDOT dyes consisting of a Zn-tetraphenylporphyrin core as a donor moiety and 3,4-ethylenedioxythiophene (EDOT) or 2-cyanoacrylic acid moiety as acceptor groups will be used to illustrate the concept of CARD to the structural engineering of new chromophores and dyes.

## 2. Working Principles of Dye-Sensitized Solar Cells (DSSCs)

Organic DSSCs mimic the charge separation process in photosynthesis in plants via photoelectrochemical (PEC) processes to form a photovoltaic (PV) device. This process has been understood for many years. The investigation of the sensitization of wide band gap semiconductor zinc oxide (ZnO) by organic dyes can be traced back to half a century ago [35,36]. The dye sensitization of titanium dioxide (TiO_2_) as a photovoltaic device can equally be traced back even to a reported usage in a US patent issued in late 1978 to Chen, Deb, and Witzk [37].

The real breakthrough in DSSCs research is credited to the seminal work of Grätzel and O’Regan in 1991 [9]. It employed a ruthenium-based dye sensitizer and achieved a reported efficiency of 7.1% PCE in a solar cell incorporating TiO_2_ [9], a relatively high power conversion efficiency for a new photovoltaic concept, which established the DSSC as a serious competitor to other solar cell technologies [38]. Until recently, a number of light-to-electrical PCEs exceeding 11% have been achieved, such as solar cells using ruthenium-based dye photosensitizers N3 [10,11], N719 [2,3,11], and black dye [12,13,14], and Carbz-PAHTDDT [25]. To achieve high efficiency, the three main components of dye-sensitized solar cells (DSSCs) (i.e., semiconductor, dye sensitizer, and redox shuttle) must be optimized [39]. The semiconductor of choice is often titanium dioxide (TiO_2_) in combination with an iodide/triiodide (3I^−^/I_3_^−^) redox couple [3,10,14,40]. In 2011, Yella et al. reported an efficiency PCE exceeding 12% [41], in which a porphyrin-based dye was co-sensitized with another organic dye sensitizer to improve the light-harvesting efficiency.

Over the past two decades, there has been a dramatic increase in research interest in DSSCs. Although significant research efforts have been made to enhance the efficiency of DSSCs, the efficiency is still lower than that of silicon-based solar cells [42] so the market for DSSC as a commercial technology has only been applied in limited numbers. An unexpected breakthrough in 2009, and remarkable results, were achieved by the introduction of perovskite structures as light-absorber materials [42,43,44,45]. The perovskite material was used as a light harvester and the cell’s redox shuttle electrolyte was replaced by an organic hole transport material, enabling an increase of the DSSC power conversion efficiency up to a record 15% [42]. However, a limitation of this new type of light absorber in DSSCs is its stability and durability issues [46,47]. As a result, organic dyes and structures for DSSCs remain an attractive area of research for further improvements in this solar cell technology.

**Figure 1:**
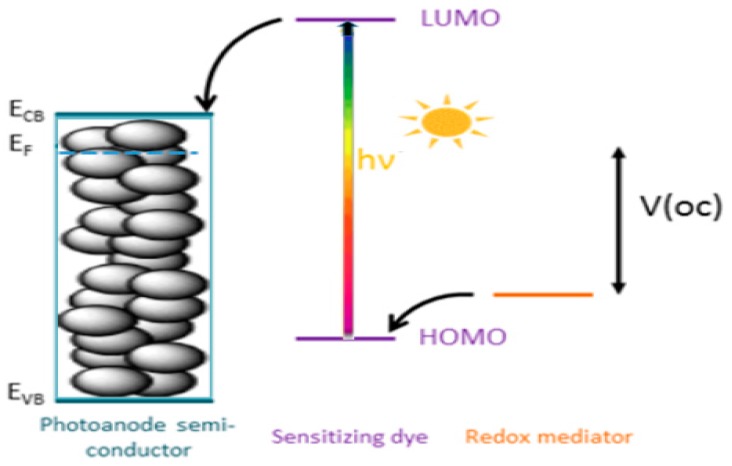
Working principle of a typical DSSC.

The primary components of DSSCs include a wide band gap mesoporous semiconductor deposited on a conducting glass substrate, a dye sensitizer, an electrolyte containing a redox couple/shuttle, and a counter electrode. The basic working principle of a DSSC and its essential properties has been well documented [28,30,48]. Depicted in Figure 1 is a schematic of the working principles of typical DSSCs.

When accepting electrons from a redox couple, such as I^−^/I_3_^−^ in acetonitrile solution, the oxidized photosensitizer is regenerated. The I^−^ anion regeneration produces a cycle as shown in Figure 1: First, when accepting electrons from a redox couple, such as I^−^/I_3_^−^ in acetonitrile solution, the oxidized photosensitizer is regenerated. Next, the oxidized product, I_3_^−^ ion, diffuses to the counter electrode (usually Pt-coated fluorine-doped tin oxide (FTO) glass plate), and finally I_3_^−^ ion is reduced back to I^−^ ion [48]. The DSSC’s performance is predominantly determined by the four electronic energy levels of the dye; that is, the energies of the HOMO and the LUMO of the sensitizer, the Fermi level of TiO_2_ (located near the conduction band level), and the redox potential of the redox couple (I^−^/I_3_^−^) in the electrolyte solution (refer to Figure 1). As indicated previously [48], photovoltage generated by the DSSCs is directly related to the HOMO-LUMO energy gap of the photosensitizer and the corresponding band gap of the TiO_2_ semiconductor. However, a smaller energy gap, Δε = HOMO-LUMO, may enable improved photocurrent generation, due to increased absorption of solar irradiation. However, for effective electron transfer, the LUMO and HOMO of the photosensitizer must reside energetically above the conduction band (CB) of TiO_2_ and below the redox couple (I^−^/I_3_^−^) [38,49,50,51]. Finally, substantial electronic coupling between the LUMO and the CB of TiO_2_ also are all factors contributing to effective electron injection and hence improvement in the overall DSSC efficiency.

Experimentally, less than optimal results at a late stage of the synthesis and device testing of dyes in DSSCs indicate an urgent need to understand the physical origin of dye properties at the molecular level, prior to experimental synthesis of target compounds [52]. As a result, CARD becomes important techniques in the discovery processes. Subsequently, CARD has been a reliable tool to design, investigate, and screen new materials prior to their synthesis, in addition to probing existing dyes [15,24,53,54,55,56,57,58,59,60,61,62,63,64,65,66,67] and finally to achieve the ultimate objective of the structural engineering of materials by rational design.

Numerous semiconductor materials can be used in dye-sensitized solar cells as an alternative to the conventional mesoporous TiO_2_ semiconductor, for example, SnO_2_ [68,69,70,71], ZnO [72,73,74], and Nb_2_O_5_ [75,76,77,78]. Further, numerous alternative redox couples to the conventional iodide/triiodide (I^−^/I_3_^−^) redox mediator, such as halogens [79,80,81], nitroxide radicals [82,83,84], sulphur-based [85,86] mediators, and transition-metal redox couples (e.g., ferrocene [87,88,89], copper (I/II) [90], cobalt (II/III) [41,91,92], and nickel (III/IV)-based complexes [93,94,95]), have been examined, reported, and summarized in previous literature [18,87,96,97]. Among them, ferrocene/ferrocenium (Fc/Fc^+^), as an effective ‘one electron outer-sphere transition-metal’ redox couple, is a kinetically fast mediator, which can work under low driving force conditions. As reported previously, this has shown promising efficiency by coupling the Fc/Fc^+^ mediator with a novel organic dye sensitizer, namely Carbz-PAHTDDT [25,87].

For DSSCs, a large class of organic dyes are available for semiconductor sensitization [18,19,98]. Organic dye sensitizers can be broadly classified into metal complexes, such as Zn-Ph dyes, [26,99,100] and metal-free organic dyes [19,101,102,103]. The more readily prepared organic dyes are usually less efficient and practical, compared to dyes that incorporate a metal center. Challenges in improving the efficiency of organic sensitizers include (a) relatively narrow absorption in the visible region, (b) shorter exciton lifetimes for excited states, (c) chemical and photochemical degradation, and (d) surface aggregation. A focus in this report is on strategies to overcome these issues using computer-aided rational design and a demonstration of enhanced dye sensitizer properties in three classes of DSSC chromophore structures.

## 3. Computational Methods

All calculations in the present study were performed using quantum chemistry methods and models. Models were based on a density functional theory (DFT) functional, using three hybrid DFT functionals, namely, B3LYP [104], PBE0 [105], and BHandH [106], combined with Polple’s polarized triple-zeta 6-311G(d) [107] basis set for the organic dyes in the present study. The PBE0 functional, a hybrid of the PBE functional with a 25% Hartree-Fock exchange term contribution, has been found to be able to produce reliable excitation energies [108,109] of organic dyes and therefore, estimates of the absorption (UV-Vis) spectra of dyes. The PBE0 functional has been, therefore, widely employed to study the coloration of many industrial organic dyes. In a benchmarking assessment of a set of over 100 organic dyes for the reproduction of experimental UV-Vis π → π* absorption spectra, the PBE0 functional, when combined with an appropriate choice of a basis set, outperformed a number of DFT functionals in previous studies [110]. As a result, both B3LYP and the PBE0 functionals were employed in this study. To reproduce solvent effects on the absorption spectra, their related molecular energy orbital levels, where required, were calculated using the polarized continuum model (CPCM) [111,112] for organic structures, such as TA-St-CA [24] and Carbz-PAHTDDT dyes [25].

The optimized geometries of the PZn-EDOT dyes using the B3LYP/6-31G* model were employed to calculate the corresponding UV-Vis spectra in solution. Here, the polarizable continuum model (PCM) in chloroform was used [26,113]. All quantum mechanical calculations were optimized to achieve a geometry with a global energy minimum, as determined by calculation of the derivative of the wavefunction, with an absence of negative frequencies observed for the calculated vibrational frequencies. Time-dependent (TD)-DFT calculations were performed for singlet states of the first 30 excitations for all organic chromophores, as above.

To analyze the charge population of the dye sensitizers, natural bond orbital (NBO) analysis was performed for selected dyes, using the NBO 3.1 program [114]. All calculations were performed using the Gaussian09 computational chemistry package [115].

## 4. Results and Discussion

### 4.1. New Dyes Based on the TA-St-CA Dye—Modification of the π-Conjugated Bridge

Reported as a highly efficient organic dye sensitizer, TA-St-CA was designed and synthesized by Suyoung et al. [116]. It achieves an overall solar-to-energy conversion efficiency of 9.1% PCE. The structure of TA-St-CA incorporates a conjugated oligo-phenylenevinylene π-bridge with an electron donor and electron acceptor moieties, i.e., D-π-A. In this section, TA-St-CA is employed to illustrate the effectiveness of CARD when applied to modify the π–conjugated bridge of the D-π-A, in order to alter the electronic structure of the archetypal dyes and effectively improve the absorption spectra of the new target dye structures.

**Figure 2:**
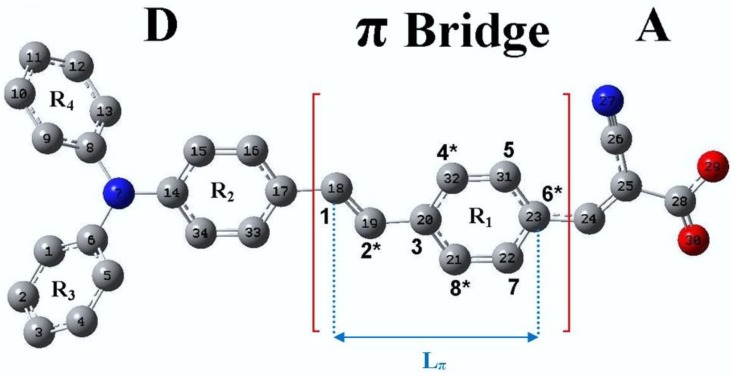
The optimized geometries and nomenclature of TA-St-CA dye. Adopted from [24] with permission.

The geometry of the TA-St-CA dye was optimized to an energetically minima with an absence of imaginary vibrational frequencies. Figure 2 depicts the optimized structure of the archetypal dye. Details of the geometric parameters of TA-St-CA can be found in [24].

**Figure 3:**
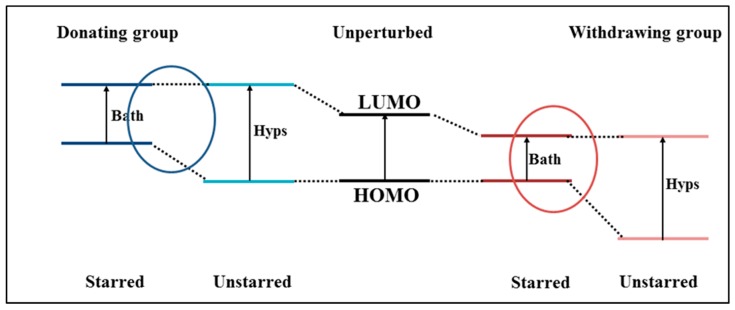
Schematic diagram of starred and unstarred orbitals in the presence of a donating and withdrawing group, according to Dewar’s rules [117]. Adopted from [24] with permission.

The new dyes’ structures can be designed following the structure–color correlation as reported as Dewar’s rules for chromophores [117]. Effectively, the π-conjugated bridge in a D-π-A chromophore exhibits alternating electronegativities along the charge-transfer direction. The energy levels of a chromophore can be changed (shifted up or down) through distinct modifications of the chemical structure of the π-conjugated bridge in comparison to the reference dye structure. For example, the π-conjugated bridge is marked by (starred and unstarred) positions as shown in Figure 2. According to Dewar’s rules, the substitution of electron-donating moieties at the starred positions will push up the energy of the HOMO, whereas substitution of electron-withdrawing groups at the unstarred marked positions will pull down the energy of the LUMO of the new dyes [118]. Depicted in Figure 3 is a representation of Dewar’s rules as a tool for modifying HOMO-LUMO states of the dye structure.

To improve light absorption, new dye candidates aim to reduce the HOMO-LUMO gap whilst concurrently red shifting the absorption spectra of the parent TA-St-CA archetypal dye chromophore. In order to achieve control of the properties of new dyes, a number of positions on the π-conjugated bridge of the TA-St-CA archetypal dye are indexed as star marked and not star marked based on Dewar’s rule, as illustrated in Figure 2. As a result, the new dye structures are obtained by chemical modifications of the backbone of the parent TA-St-CA dye through substitutions around these positions with various electron-donating (ED) moieties and electron-withdrawing (EW) moieties using Dewar’s guidelines. Two ED groups (NH_2_ and N(CH_3_)_2_) and one EW group (CN) are substituted at the appropriate positions systematically one at a time to produce a number of new dye candidates, which are summarized in Table 1. For the complete structures of the new dyes, refer to Table S1 in the supplementary information in [24].

An investigation of absorption properties, with data represented as UV-Vis spectra, is an appropriate technique to reveal trends in the influence of changes to chromophore centers of a molecular structure and gain an understanding of structure–property relationships. Chromophores containing moieties with π-electrons/lone electron pairs often absorb in the visible region (200 to 800 nm). Hence, structural changes via π-electrons in the chromophores through the addition/substitution of chemical structurally features can be probed experimentally using UV-Vis spectroscopy. In silico, the UV-Vis spectra of different chromophores (new candidate structures) using an appropriate theoretical model can be applied to the parent TA-St-CA dye to simulate the UV-Vis spectra of new dye derivatives. The geometries of the new dye derivatives were optimized using the same DFT functional/basis set in vacuum and in a solvent dielectric medium. Subsequently, absorption UV-Vis spectral profiles were calculated (see the experimental section) for the lowest 30 spin-allowed singlet-singlet transitions [32,99].

**Figure 4:**
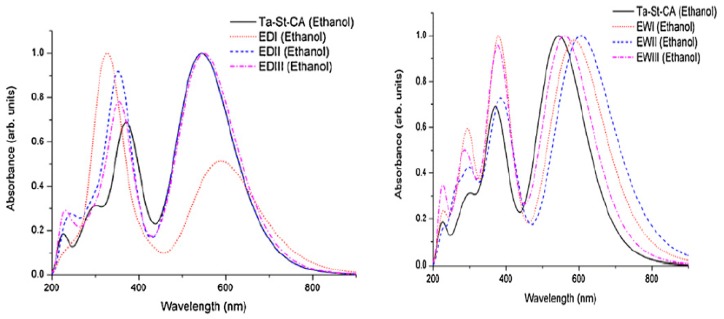
The calculated UV–Vis absorption spectra of the parent TA-ST-CA dye and the computer-designed new dyes ED-I to ED-III and EW-I to EW-III in ethanol solution using the TD-DFT calculations. Adapted from [24] with permission.

Structural fragments and positions changed to the parent compound are summarized in Table 1 and absorption profiles are depicted in Figure 4. It is seen that the UV-Vis spectrum of TA-St-CA consists of two major spectral peaks [24,34] at λ = 358 nm (I) in the ultraviolet region and λ = 501 nm (II) in the mid-visible region, in agreement with the literature [119]. The most intense absorption band is observed at λ = 501 nm (f = 1.0777) corresponding to a transition (excitation) of HOMO → LUMO of TA-St-CA dye, whereas the weaker absorption band near λ = 358 nm (f = 0.8699) is a summation of two major transitions around the frontier orbitals: HOMO-1 → LUMO (84%) and HOMO → LUMO + 1 (15%).

**Figure 5:**
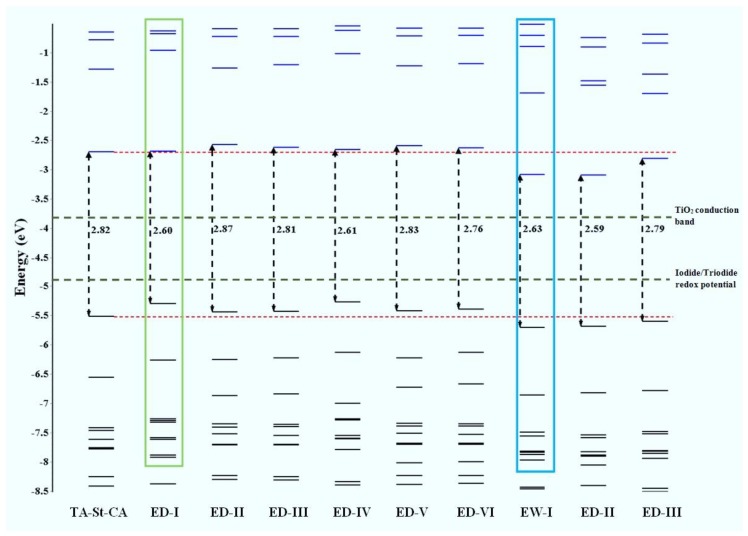
Calculated frontier orbital energies using the PBE0/6-31G* model [23]. Refer to Table 1 for the new dye nomenclatures and structures. Adopted from [24] with permission.

As indicated above, achieving control of the spectral absorption of new dyes requires an understanding of the structural–absorption relationships of the chromophores. Figure 5 provides the frontier orbital energy diagrams of the parent TA-St-CA dye and structurally engineered candidate compounds [34,67]. For the parent structure, orbital energies of the HOMO and LUMO were calculated to be −5.51 and −2.69 eV, respectively, with an energy gap of the HOMO and LUMO of 2.82 eV. In general, substitution by varying the functional groups around the center to form new candidates does not impart a significant reduction in the energy gap of the HOMO-LUMO in comparison to the parent TA-St-CA dye. However, a greater reduction of the HOMO-LUMO gap of the new dyes can be achieved through strategic altering of either the HOMO or LUMO energies. For example, the ED substitutions on the 2*, 4*, and 8* positions of TA-St-CA, and alternatively, the EW substitutions in positions, such as 5 (in new dyes EW-I, EW-II, and EW-III), results in a reduction of the HOMO-LUMO energy gap. The latter is achieved by pulling both the HOMO and the LUMO energies down, which is clearly show in Figure 5. Hence, ED group substitutions at positions close to the donor (D) section of the parent dye can be more effective for the new dyes in DSSCs [24,117]. This approach can be applied as CARD to design other chromophore structures.

### 4.2. CARD for Enhanced Structures from Carbz-PAHTDDT Dye Sensitizer

In a recent report, a chromophore, Carbz-PAHTDTT (S9), demonstrated the use of a D-π-A structure in an alternative DSSC architecture with a Fc/Fc^+^ redox couple [88], motivating theoretical investigation of desirable properties using rational design strategic of the parent dye S9. Assessing the performance of the S9 dye structure and derived structures provides an understanding of electronic structural properties and potential pathways to improved dye candidates using the CARD [25].

**Figure 6:**
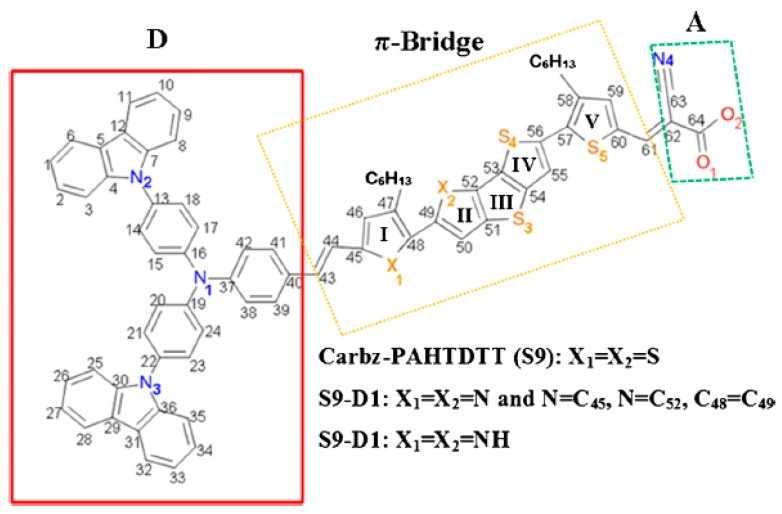
Schematic diagram of the parent dye Carbz-PAHTDTT (S9) and new dyes S9-D1 and S9-D2. Adopted from [25] with permission. Dye S9: X_1_ = X_2_ = S. Dye S9-D1: X_1_ = N = C_45_, X_2_ = N = C_52_ and dye S9-D2: X_1_ = X_2_ = NH.

The backbone molecular structure (hydrogens omitted) of the D-π-A dye of Carbz-PAHTDTT (S9) [88] is depicted in Figure 6, where the structure consists of the typical D-π-A components. A dithienothiophene (DTT) unit forms the central core of the π-conjugated bridge for the parent S9 dye. The electron donor moiety (D) is non-coplanar, which has been shown to enhance the thermal stability of the dye sensitizer by reducing the intermolecular distance and aggregation. The π-bridge contains a number of five-membered heterocyclic rings, which are labelled as I, II, III, IV, and V in Figure 6. To provide extended conjugation in the linker [120], two hexanyl (C_6_H_13_)-substituted thiophene rings (i.e., 3-hexylthiophene, rings I and V in Figure 5) are added to the π-conjugated bridge of the S9 dye. The hexanyl chains facilitate two possible isomers, one for cis-S9 anther for trans-S9 dyes. The inclusion of long hexyl chains suppresses the aggregation of the dye molecules, which contributes to extended electron lifetime (τ) [121]. Quantum mechanical calculations reveal that the cis-S9 conformer is more energetically preferred, with a total energy of approximately 4.6 kJ⋅mol^−1^ less than the total energy of the trans-S9 conformer. As a result, the present CARD study focuses on the cis-S9 conformation. The acceptor (A) moiety of the parent S9 dye includes a cyano group as an electron-withdrawing group and the carboxyl group serves as an anchoring unit to contact the dye to the TiO_2_ surface, as shown in Figure 6 [25].

An objective of developing new dye candidates is to extend the light absorption of the dye beyond the UV-vis region to the near infrared (NIR) region by modification of its electronic structure. This, in effect, is achieved by minimizing the HOMO-LUMO energy gap of the dye sensitizer. For D-π-A chromophores, the extensively conjugated π-electrons of the core structure are often the focus in structural design. Here, we investigated rational modification of the π-bridge of the parent S9 dye, in order to generate two new dye structures. As a result, modification of the π-linker of the cis-Carbz-PAHTDTT (S9) parent structure to form two new derivatives, S9-D1 and S9-D2, was conducted. The new candidate dyes, S9-D1 (Figure 6, where X_1_ and X_2_ = N) and S9-D2 (Figure 6 where X_1_ and X_2_ = -NH) [25], are depicted in Figure 6 alongside the optimized structures of the parent Carbz-PAHTDTT (S9) dye.

**Figure 7:**
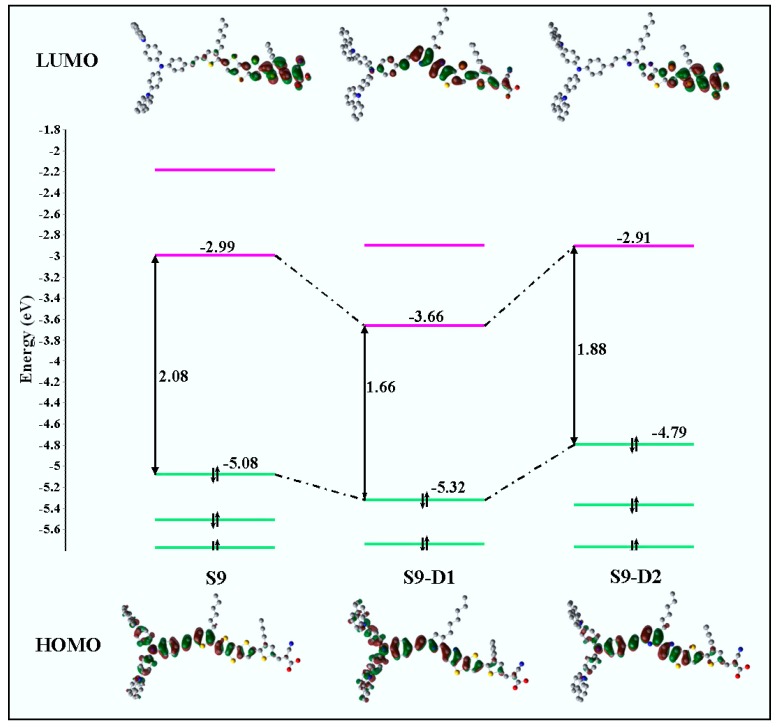
Calculated valence MO energy levels (HOMO and LUMO orbitals) of the dyes, i.e., S9, S9-D1, and S9-D2 dyes. Note: a different model, the CPCM-B3LYP/6-311G(d)//CPCM-PBE0/6-311G(d) model, was also employed [25] in the DCM solution for comparison reasons. Adopted from [25] with permission.

Such in silico chemical modification of the core chromophore results in reordering of the electronic structures of the new chromophores, directly inducing change in the frontier orbital energy levels through the π-conjugated bridge, which in turn changes the spectral absorption profiles for transitions between the occupied and virtual orbitals of the dyes. The measured energies, such as the orbital energies of HOMO, LUMO, and energy gap of HOMO-LUMO of the parent S9 dye in dichloride methane (CH_2_Cl_2_, DCM) solution, were reported [88] as −5.08, −2.97, and 2.11 eV, respectively. Depicted in Figure 7 is a comparison of the calculated outer valence molecular orbital energies of the new candidate dye with respect to the parent S9 dye in DCM solution. The HOMO-LUMO energy gaps of the dyes are also provided and marked in the figure. As shown in Figure 7, there was an apparent decrease in the HOMO-LUMO energy of the new S9-D1 and S9-D2 dyes compared to the parent S9 reference structure. These were decreased from 2.08 eV in S9 to 1.66 and 1.88 eV in S9-D1 and S9-D2, respectively.

The new dye structures S9-D1 and S9-D2 both achieved the objective of reduced HOMO-LUMO energy gaps, yet this was via different mechanisms. For example, for S9-D1, lowering of the LUMO energy from −2.99 (S9) to −3.66 eV (S9-D1), with a concurrent minor change in the energy of the HOMO of S9-D1, this was lowered from −5.08 (S9) to −5.32 eV (S9-D1) [25]. For S9-D2, the significant change was achieved by an increase of the HOMO energy level from −5.08 (S9) to −4.79 eV (S9-D2). The LUMO energy remains relatively unperturbed from −2.99 (S9) to −2.91 eV (S9-D2) [25,88]. As a result, the current investigation demonstrates that the substitution of an electron-donating group (N) in the π-linker contributes to lowering the LUMO of the new dye S9-D1 from the parent dye (S9), whereas substitution of an electron-withdrawing group (NH) in the π-linker of S9 can effectively equally increase the HOMO of the new dye S9-D2.

**Figure 8:**
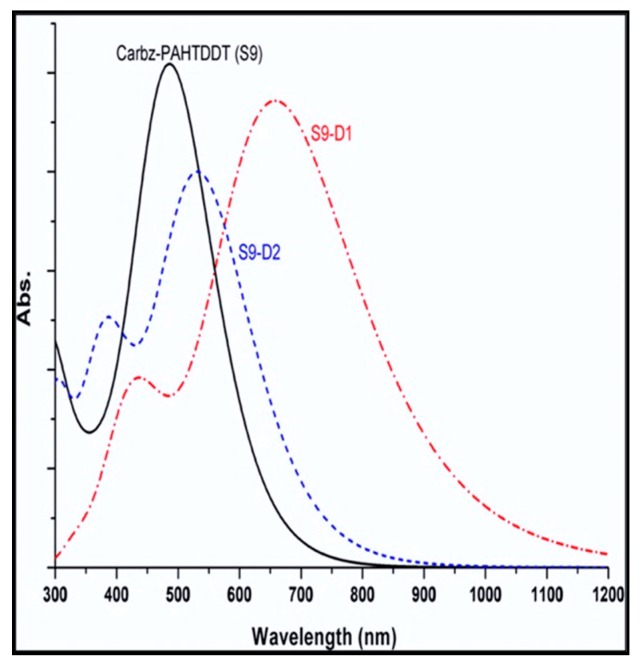
Comparison of the calculated UV-vis spectra of three dyes S9, S9-D1, and S9-D2 dyes in DCM solution. The CPCM-B3LYP/6-311G(d)//CPCM-PBE0/6-311G(d) model in DCM solution was employed [25]. Adopted from [25] with permission.

Presented in Figure 8 are simulated absorption profiles of the three dyes examined in this study: S9-D1 and S9-D2 and the parent structure S9 as a reference [25]. Depicted in the figure are one major and two minor absorption bands of the S9 dye centered at λ_1_ = 668 nm, λ_2_ = 540 nm, and λ_3_ = 488 nm, respectively. It should be noted that due to the simulated profiles’ distribution, not all three calculated peaks are discernible in Figure 8. Changes in HOMO-LUMO transitions are apparent for the new dye S9-D1, compared to that of the parent S9 dye, leading to a red-shift of λ_1_ in the spectrum. Further, a significant red-shift of the λ_2_ and λ_3_ bands is also observed for S9-D1 with respect to the position of bands in the S9 dye. For example, the absorption λ_1_ band in the simulated spectrum of the new dye S9-D1 is significantly red-shifted compared to the parent Carbz-PAHTDTT (S9) dye with broadening of the spectral band (when represented as an absorption profile). This is intensified in the S9-D1 dye, which exhibits significant red (bathochromic) shift and broadening of the absorption profile, effectively enhancing light harvesting—an important criterion for effective chromophores as a surface sensitizer in DSSCs. With respect to S9-D2, there is a minor bathochromic shift of 44 nm in simulated bands for λ_1_ and λ_3_ with respect to S9. Conversely, an undesirable hypochromic shift (i.e., blue-shifted to shorter wavelengths) of −32 nm for the λ_2_ spectral band, however, was also obtained for S9-D2.

Table 2 summarizes the calculated spectral parameters of the three dye structures using varied DFT functionals. Considering the data obtained for the parent S9 dye, with respect to the simulation of spectral profiles, DFT with the TD-BHandH functional more closely reproduces the major spectral band at λ_1_ = 490 nm compared to the experiment, λ_1_ = 491 nm. Further, deeper frontier orbital transitions represented by minor bands centered at λ_2_ = 402 nm (experiment 426 nm) and λ_3_ = 360 nm (experiment 330 nm) are also closely reproduced by the same model [88]. For the purposes of using CARD to guide structural engineering of new dyes, the exchange energy component is important in DFT functionals, in order to accurately reproduce the simulated absorption profiles of reference and candidate chromophores. That is, the use of B3LYP and PBE0 hybrid functionals is less accurate when compared to the BHandH functional. The spectral band positions of the dyes are more closely represented, due to less accurate calculation of virtual orbital energies for such dyes, as reported previously for related structures [122,123].

### 4.3. CARD for Enhanced Structures from Zinc Porphyrin Macrocyclic Dyes

Porphyrin and derivative chromophore structures provide a highly flexible class of compounds for the development of panchromatic sensitizers [100,125,126,127]. Free porphyrin chromophore is well known for its four-orbital (4Orb) model [128,129]. It results in four absorption bands (Q bands) from red to purple, which appear in the visible region of 500 to 700 nm of the absorption spectra [99,125,127]. A very sharp but intense band (B band) in the Soret (UV) region at ca. 400 nm has intrinsically strong light absorption. Yet, there is a lack of significant absorption in the spectral region between these two bands, i.e., the Q-bands and Soret bands. It has been reported that the porphyrin macrocycle forms superior organic dyes using a co-sensitization method (YD2-o-C8) [130] in DSSCs, in which a cobalt-based electrolyte is able to achieve a comparable photoelectric conversion efficiency of 11% PCE compared to the conventional ruthenium-based N719 dye [41]. As a result, metalloporphyrin compounds, such as molecularly engineered metalloporphyrin, a Zn-porphyrin-based dye (coded as SM315), have previously been reported with impressive properties for DSSCs applications [125]. This Zn-based porphyrin (SM135) dye equally possesses a prototypical structure of D–π-A and exhibits improved light-harvesting properties, achieving 13% PCE [131].

Further studies have shown that the Zn-porphyrin-based SM315 sensitizer [127] exhibits a vivid green coloration due to an absence of absorption between 500 and 600 nm [113,115,125,127,131,132]. In this study, we investigated the rational for chemical modifications of a reference Zn-porphyrin compound in order to identify the overall light absorption for new derivative dye structures [26]; that is, to rationally modify the Zn-porphyrin structure in order to reorder the four frontier orbitals of the porphyrin structure affecting a shift in the energy for transitions, and effectively tuning spectral absorption.

**Figure 9:**
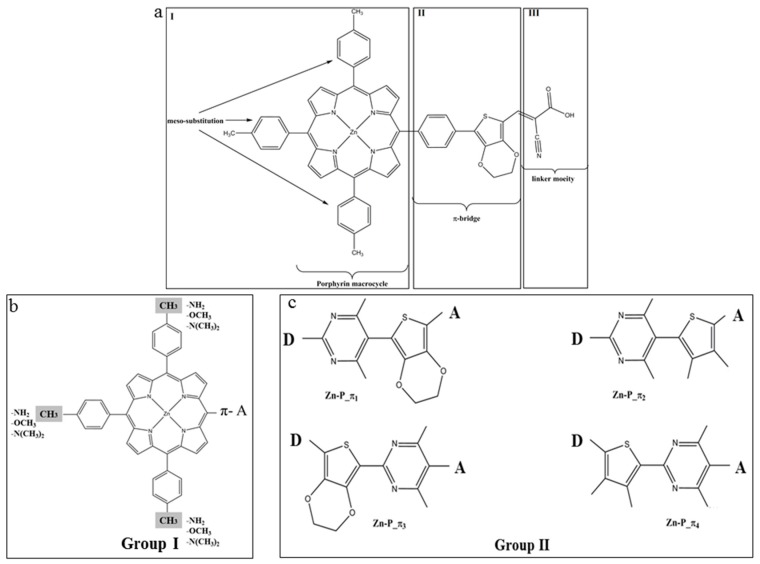
The chemical structure of (**a**) PZn-EDOT and two groups of derivatives (**b**) Group I to modify the meso-positions of porphyrin moiety; and (**c**) Group II to modify the π-linker [26]. Royal Society of Chemistry (RSC Adv.) is acknowledged.

The donor moiety of the reference parent PZn-EDOT dye, i.e., Zn-tetraphenylporphyrin, is depicted in Figure 9a. It is a widely utilized supramolecular chromophore building block [133]. The strong visible light absorption bands of Zinc-porphyrin derivatives are a result of π–π* transitions from the closely spaced HOMO and LUMO frontier valence orbital manifold [132,134]. Transitions between frontier orbitals are able to produce a high energy S_2_ excited state (Soret band) at approximately 420 nm with a large oscillator strength, as well as a lower energy S_1_ excited state (Q band) with a diminished oscillator strength between 500 and 650 nm [132,134]. Importantly, the Zn porphyrin derivative was synthesized recently by Xiang et al. [135], i.e., 2-cyano-3-(3,4-ethylenedioxy-5-(4-(10,15,20-tris(4-methylphenyl))-porphyrina-tozinc(II)yl)-phenyl)-thienyl acrylic acid (Pzn-EDOT), see Figure 9a.

In the original D-π-A structure of PZn-EDOT, the Zn porphyrin macrocycle acts as an electron donor (D) moiety, a cyanoacrylic acid group as an electron acceptor (A) moiety, and 3,4-ethylenedioxythiophene (EDOT) as a π-bridge. In comparison, the SM315 dye exhibits significant broadening of the Soret and Q-band absorbance, leading to an enhancement in absorption in the green region [125]. In order to redshift (bathochromic shift) the absorption spectrum, it is necessary to decrease the HOMO-LUMO energy gap of the new dyes through chemical changes of the structures [24]. Strategies to enhance spectral absorbance include substitution of the donor (D) moiety, the π-bridge, or the acceptor moiety (A) of the D-π-A structure, respectively. In an earlier study of CARD of the TA-St-CA dyes [24] (above in Section 4.1), chemical modifications focused on the π-bridge according to Dewar’s rules [34,128], using electron-donating (ED) fragments and electron-withdrawing (EW) fragments. We found that chemical modifications of the parent TA-St-CA dye using ED-substitutions along the backbone of the π-bridge exhibit advantages over the EW-substitution, for decreasing the HOMO-LUMO energy gap in new dye structures [24]. In the new dyes, the HOMO energies of the new dyes upward shift more apparently than the downward shift of the LUMOs [24]. The donor moiety (metalloporphyrin) of the reference Pzn-EDOT dye was modified using ED-fragments—which produce Group I dyes and the π-bridge—which produces Group II dyes, as indicated in Figure 9 [26].

When bonding with a metal atom, such as Zn, the UV-Vis spectrum of Zn-porphyrin essentially retains the pattern, with a sharp intense Soret B band at approximately 400 nm in the near UV region and a couple of weak Q bands in the visible region between 500 and 700 nm, which are responsible for the red to purple colors [136]. The quantum mechanically calculated UV-Vis spectrum of the original dye (PZn-EDOT) in the chloroform solution agrees well with the measured spectra in the same solvent [26]. For example, the spectrum exhibits a strong Soret band at 423 nm (S0 → S2) and moderate Q band at 550 nm in the measurement [135]. The simulated spectrum of PZn-EDOT shows two major bands, with a strong Soret band at 432 nm and the less intense Q band at 585 nm, respectively. 

**Figure 10:**
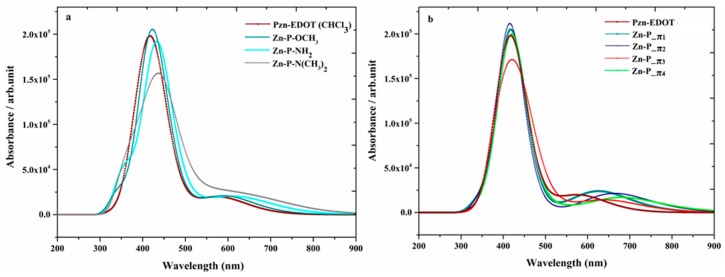
Simulated absorption profiles of modified zinc porphyrin dyes using (**a**) Group I; and (**b**) II moieties in CHCl_3_ solution [26]. Royal Society of Chemistry (RSC Adv.) is acknowledged.

Depicted in Figure 10, for Group I dyes (a) and Group II dyes (b), is a comparison of the quantum mechanically calculated UV-Vis absorption spectra of the new dyes together with the parent PZn-EDOT [135,137] in chloroform (CHCl_3_) solution. The spectra of the new dyes in this figure exhibit both Q-bands and a strong Soret band and indicate an apparent bathochromic shift (red shifted) or broadening compared to the reference PZn-EDOT dye. As noted in previous reports [138], engineering structural changes perturb the core porphyrin macrocyclic structure through various chemical modifications, with the intent to impart an effect on the intensity and absorption position of the Q band and shift absorption to the desired spectral region. For example, insertion of *aza* groups at the meso positions of the porphyrin leads to contraction of the macrocyclic center; whereas replacing nitrogen-containing moieties at meso carbon positions significantly intensifies the oscillator strength of the higher energy shoulder for transitions of the Q-band without a significant change in the apparent band position [128,138].

Summarized in Table 3 are calculated parameters of the major spectral parameters of the new macrocyclic structures [26]. As shown in the data, all new structures exhibit split Soret band transitions, which has been widely reported for Zn-porphyrin assemblies (tetramers) and also structures, such as SM315 dye [125]. Near the region of 400 nm, the Soret band is split into two major excitations, with one blue-shifted below 430 nm and another red-shifted above 430 nm. The magnitude of the split in transitions ranges from 9 to 42 nm for the multiple candidate macrocycles. For example, the Soret band for Pzn-EDOT‒NH_2_ splits to 438 (1.84) and 429 nm (0.94) in chloroform (CCl_4_) solution. Experimentally, split transitions are observed in the Soret band for the UV-vis spectra of the high-performance metalloporphyrin SM315. The observed absorption maxima at 440 and 454 nm represent a 14-nm separation in the Soret band [125,131].

Alternatively, structures represented in Group II, such as Zn-P-π3, exhibit similar band splitting at 451 (1.56) and 409 nm (1.39) in the calculated absorption profile. Closer inspection of the candidate structures in Group II, Zn-P_π1, and Zn-P_π3 dyes, the Soret-band positions in the spectra are 427 (2.36) and 407 nm (1.40) for Zn-P_π1, and 451 (1.56) and 409 nm (1.39) Zn-P_π3, which are all red-shifted. As a result, substitution by moieties, such as EDOT and pyrimidine (Py), become more attractive for solar cell applications than other Group II fragments as they provide preferred spectral absorption properties. Further, the substitution position of chemical fragments also show apparent differences. For example, in both Zn-P_π3 and Zn-P_π1 dyes, the π-bridges of both dyes consist of EDOT and Py monomers, yet the π-bridge connections are either through the thiophene or pyrimidine groups, which impart observable differences when the substitution position is modified. In the former (Zn-P_π3), the π-bridge is provided by conjugation through –EDOT-Py and in the latter (Zn-P_π1), the π-bridge is reversed as –Py-EDOT. Importantly, inversion of this molecular fragment imparts a quite different magnitude of the red shift of electronic transitions in the region of the Soret band. This band is centered around 451 nm for Zn-P_π3 with the π-bridge of –EDOT-Py; the same band is further hypsochromically (blue) shifted to 427 nm for the Zn-P_π1 dye with the π-bridge inverted to –Py-EDOT. Therefore, structures, such as Zn-P_π3, are more attractive chromophores for solar cell applications than those resulting from Zn-P_π1 dye, which is in agreement with previous reports on molecular properties, including the HOMO-LUMO energy gap and dipole moment, etc. [26].

**Figure 11:**
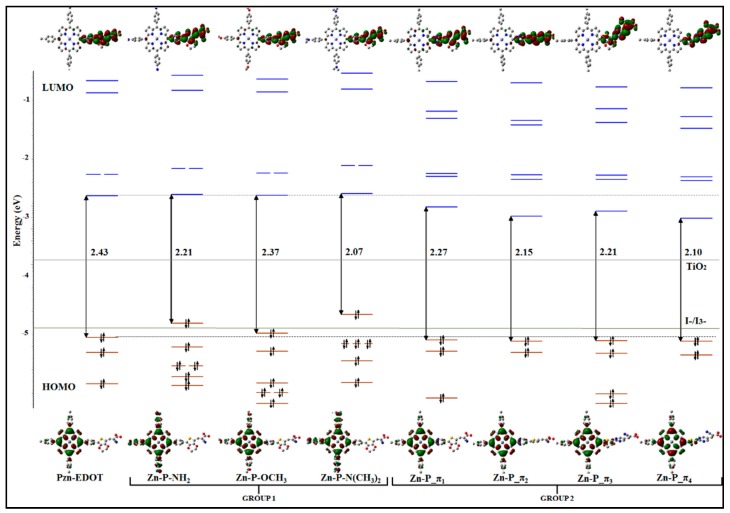
Schematic representation of frontier molecular orbitals of PZn-EDOT and derivative macrocycle structures, from Group I and Group II moieties (see description in main text and molecular fragments in Figure 9) [26]. Depictions indicate Gouterman’s classical porphyrin four orbital (4Orb) model. Royal Society of Chemistry (RSC Adv) is acknowledged.

In Figure 11, the orbital energy diagrams of the frontier orbitals for PZn-EDOT macrocycle and derivative structures are depicted, with the geometries energies all calculated in chloroform solution [26]. The calculated orbital energies of the parent PZn-EDOT dye are in good agreement with Gouterman’s porphyrin four-orbital (4Orb) model [128,129]. In the four orbitals of the parent dye, HOMO and HOMO-1, which are doubly occupied and the LUMO and LUMO+1, which are virtual (unoccupied), are tightly clustered in energy as represented in the diagram. These four frontier orbitals are relatively separated in energy from deeper orbitals of the same compound, hence dominant transitions impart an influence on the UV-Vis-NIR region of the absorption profile. Electronic transitions of PZn-EDOT are predominately from occupied orbitals, such as HOMO and HOMO-1, into the virtual (unoccupied) orbitals of LUMO and LUMO + 1, with the resultant spectra representative of the parent dye. Structural engineering of macrocyclic compounds using CARD necessitates an imparting of the electronic influence from molecular fragments to destabilize primary transitions within the four frontier orbitals and increase participation of low-lying orbitals, occupied or unoccupied, that are energetically closer to frontier orbitals in spectral transitions. In the example of the new derivatives of PZn-EDOT, the frontier orbital manifold has been appropriately influenced by differing molecular fragments as indicated by changes in the energy level of the HOMO and LUMO states of the four frontier orbitals as shown in Figure 11. As a result, an increased number of deeper orbitals are accessible in the macrocycle derivatives in comparison to the parent PZn-EDOT dye, effectively broadening the spectral absorption window as shown for the calculated profiles in Figure 10.

Further, to extend the spectral absorption for DSSC applications, new candidate structures should target decreasing the HOMO-LUMO energy gap and tuning the occupancy and accessibility of low-lying virtual orbitals. This can be achieved by increasing or decreasing the energy of the electronic states of the HOMO and LUMO states surrounding the frontier orbital manifold, respectively. As shown in Figure 11, the four-orbital manifold of the new derivative macrocycle structures has enabled a decrease in the HOMO-LUMO energy gap of the reference parent PZn-EDOT dye (from 2.43 eV), using molecular fragments from both Group I or Group II moieties. For example, for structurally dissimilar derivatives, Group I (Zn-P-NH_2_) and Group II (Zn-P-π_3_) are isoenergetic with a HOMO-LUMO energy gap of 2.21 eV, yet they are not isoelectronic, with significant differences in the deeper states surrounding the frontier molecular orbital manifold.

## 5. Conclusions

Computer-aided rational design (CARD) is an invaluable methodology for structural engineering of new molecular compounds, designed in silico, with multiple pathways available to improve the spectral absorption properties of chromophores through molecular modifications using chemical fragments to impart electronic structural influence. This mini review demonstrates how CARD can enhance desirable optical properties, such as broadening and red-shifting the spectral absorption of established D-π-A organic dyes in DSSC applications. Through computer-aided rational molecular modification, it is conceivable to develop new and more effective organic chromophores with desirable properties with a reduced opportunity-cost ratio compared to matrix screening of synthetic analogues in wet chemistry laboratories. This was illustrated through the process of modifying the conjugate π-bridge of the parent TA-St-CA dye based on Dewars’ rules for chromophores. It was demonstrated that decreasing the energy of the HOMO-LUMO gap in new candidate structures through the substitution of electron-donating (ED) fragments exhibits advantages over a similar substitution with electron-withdrawing (EW) fragments, although imparting a minimal impact on the calculated absorption profile [24]. Further, investigation of the organic dye, Carbz-PAHTDTT (S9), through rational modification of the π-bridge resulted in two new chromophores, S9-D1 and S9-D2, using -N and -NH substitutions, respectively. Two new dyes (S9-D1 and S9-D2) exhibit desirable red-shifting and broadening as indicated by the calculated absorption profile [25]. Finally, using the example of a Zn-centred metalloporphyrin macrocycle, alteration of the stable four-orbital frontier manifold of the porphyrin structure resulted in new candidate dyes with broader apparent absorption suitable for DSSC applications. Structural engineering via molecular fragment modifications of the metalloporphyrin donor (D) moiety and the conjugate π-bridge enabled a decrease in the HOMO-LUMO energy gap and broadened the calculated absorption profile, effectively enhancing the light-harvesting capacity of the new chromophores [26]. This was illustrated by modifications to the π-bridge with dyad moieties, such as pyrimidine (Py), thiophene (THn), and EDOT, resulting in a series of Group II structures, which are more attractive for DSSC applications [24,26]. This mini review outlines the positive attributes of the application of CARD principles for the design and structural engineering of new chromophores for targeted applications, including DSSC devices. The studies outline the necessity of understanding structure–property relationships as a step closer to control in the design of functional materials. It is articulated that when CARD combines with machine learning (ML) and artificial intelligence (AI), there will be significant leap forward in organic DSSCs in the energy sector [139].

## Figures and Tables

**Table 1:** New dyes obtained from chemical modifications of TA-St-CA along the π-bridge using the ED (electron donating) and EW (electron withdrawing) substitutions (backbone only) based on Dewar’s rule [24].

Label	Structure *	Substitution Type	Chemical Fragment	Position
**ED-I**	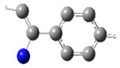	Electron Donating	NH_2_	2*
**ED-II**	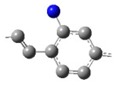			4*
**ED-III**	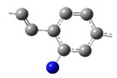			8*
**ED-IV**	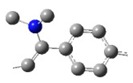		N(CH_3_)_2_	2*
**ED-V**	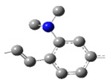			4*
**ED-VI**	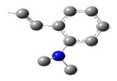			8*
**EW-I**	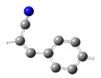	Electron Withdrawing	CN	1
**EW-II**	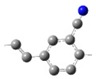			5
**EW-III**	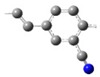			7

* Only the π-bridge fragment in the structure in Figure 2 is shown in this table. Adopted from [24] with permission.

**Table 2:** Comparison of spectral parameters of the most intense absorption peak positions of the original and derivative dyes, S9, S9-D1, and S9-D2.

	Carbz-PAHTDTT (S9)			S9-D1		S9-D2	
Method ^(a)^	TD-B3LYP	TD-BHandH	Exp.^(b)^	TD-BHandH	Δλ ^(c)^	TD-BHandH	Δλ ^(d)^
λ_1_ (nm)	668	490	491	662	172	535	45
λ_2_ (nm)	540	402	426	528	126	394	−8
λ_3_ (nm)	488	360	330	440	80	374	14

^(a)^ All TDDFT calculations were performed in DCM solution using the CPCM solvation model. Adopted from [25] with permission. ^(b)^ See supplementary information of [25,88,124]. ^(c)^ ∆λ = λ(S9 − D1) − λ(S9), method = TD − BHandH. ^(d)^ ∆λ = λ(S9 − D2) − λ(S9), method = TD − BHandH.

**Table 3:** Comparison of experimental and theoretical frequencies in vacuum and CCl_4_ solution for the Soret and Q-bands of new macrocyclic structures based on the reference Pzn-EDOT structure [26]. *

Zn-Porphyrin	VACUUM	CCl_4_
	Soret band, Q-band	Soret band, Q-band
**Pzn-EDOT (exp)**	-	423(2.55), 550(0.17)
**Pzn-EDOT(theo)**	415(1.98), 570(0.22)	432(2.26), 585(0.32)
**Group I**		
**Zn-P-NH_2_**	420 (1.72), 409 (0.77), 622(0.21)	438(1.84), 429(0.94), 642(0.28)
**Zn-P-OCH_3_**	393(1.11), 372(1.00), 585(0.23)	433(2.33), 411(1.46), 601(0.33)
**Zn-P-N(CH_3_)_2_**	429(0.97), 405(0.87), 658(0.19)	449(1.04), 422(1.01), 685(0.25)
**Group II**		
**Zn-P_π_1_**	413(2.07), 392(0.90), 643(0.34)	427(2.36), 407(1.40), 629(0.43)
**Zn-P_π_2_**	416(1.03), 401(1.03), 687(0.31)	422(2.29), 410(1.38), 666(0.38)
**Zn-P_π_3_**	437(1.11), 392(0.77), 637(0.18)	451(1.56), 409(1.39), 650(0.24)
**Zn-P_π_4_**	419(1.97), 390(1.05), 680(0.20)	432(2.22), 409(1.28), 686(0.30)

* Oscillator strength is shown in parentheses. Royal Society of Chemistry (RSC Adv.) is acknowledged.
